# Platelet-rich plasma for the treatment of diabetic foot ulcer: a systematic review

**DOI:** 10.3389/fendo.2023.1256081

**Published:** 2023-11-18

**Authors:** Hong OuYang, Yi Tang, Fan Yang, Xin Ren, Jing Yang, Hongyi Cao, Yifan Yin

**Affiliations:** ^1^ Geriatric Diseases Institute of Chengdu, Department of Endocrine and Metabolism, Chengdu Fifth People’s Hospital (The Second Clinical Medical College, Affiliated Fifth People’s Hospital of Chengdu University of Traditional Chinese Medicine), Chengdu, China; ^2^ Department of Nephrology, Chengdu Third People’s Hospital, Chengdu, China

**Keywords:** diabetes, platelet-rich plasma, diabetic foot ulcer, therapeutic index, preparation condition

## Abstract

**Background:**

With the increasing incidence of diabetes, diabetic foot ulcer(DFU) has become one of the most common and serious complications in people with diabetes. DFU is associated with significant morbidity and mortality, and can also result in significant economic, social and public health burdens. Due to peripheral neuropathy, peripheral vascular disease, hyperglycemic environment, inflammatory disorders and other factors, the healing of DFU is impaired or delayed, resulting in the formation of diabetic chronic refractory ulcer. Because of these pathological abnormalities in DFU, it may be difficult to promote wound healing with conventional therapies or antibiotics, whereas platelet-rich plasma(PRP) can promote wound healing by releasing various bioactive molecules stored in platelets, making it more promising than traditional antibiotics. Therefore, the purpose of this systematic review is to summarize and analyze the efficacy of PRP in the treatment of DFU.

**Methods:**

A literature search was undertaken in PubMed, CNKI, EMB-ASE, the Cochrane Library, the WanFang Database and the WeiPu Database by computer. Included controlled studies evaluating the efficacy of PRP in the treatment of diabetic foot ulcers. The data extraction and assessment are on the basis of PRISMA.

**Results:**

Twenty studies were evaluated, and nineteen measures for the evaluation of the efficacy of PRP in DFU treatment were introduced by eliminating relevant duplicate measures. The efficacy measures that were repeated in various studies mainly included the rate of complete ulcer healing, the percentage of ulcer area reduction, the time required for ulcer healing, wound complications (including infection rate, amputation rate, and degree of amputation), the rate of ulcer recurrence, and the cost and duration of hospitalization for DFU, as well as subsequent survival and quality of life scores. One of the most important indicators were healing rate, ulcer area reduction and healing time. The meta-analysis found that PRP was significantly improve the healing rate(OR = 4.37, 95% CI 3.02-6.33, P < 0.001) and shorten the healing time(MD = -3.21, 95% CI -3.83 to -2.59,P < 0.001)of patients with DFU when compared to the conventional treatment, but there was no significant difference in reducing the of ulcer area(MD = 5.67, 95% CI -0.77 to 12.11,P =0.08>0.05 ).

**Conclusion:**

The application of PRP to DFU can improve ulcer healing rate and shorten ulcer healing time, but more clinical data are needed to clarify some efficacy measures. At the same time, a standardized preparation process for PRP is essential.

## Introduction

The global incidence of diabetes is increasing rapidly. The International Diabetes Federation(IDF) estimates that the prevalence of diabetes will increase from 10.5%(536.6 million people in the 20-79 age group) in 2021 to 12.2%(783.2 million people in the 20-79 age group) by 2045 (1). It is projected that nearly half of adults (44.7 percent; 239.7 million people in the 20-79 age group) do not know they have diabetes, and people may be more susceptible to microvascular and macrovascular complications in an asymptomatic diabetic state (2).

Diabetic foot ulcer (DFU) is one of the most common and serious complications in patients with diabetes (3–6) and is characterized by complex management, high morbidity, and high mortality (7). The annual incidence of diabetic foot around the world ranges from 9.1 million to 26.1 million (8, 9), with a global prevalence of about 6.3%, which mostly occurs in patients with type 2 diabetes, the elderly, and people with a longer duration of diabetes (10). In China, the prevalence of DFU is increasing with the increase of the incidence of diabetes year by year. According to statistics, the incidence of DFU in people over 50 years old in China is as high as 8.1% (3, 11). DFU continue to be an important cause of hospitalization in patients with diabetes and form the basis of 40-70% of diabetic non-traumatic lower limb amputations (12, 13).

Relevant reports have also shown that nearly 88% of lower leg amputations are associated with diabetic foot ulcers (14). In addition, the global annual cost of DFU treatment and amputation is approximately US $10.9 billion (15), and the cost of DFU treatment in China will rise from the current US $4.9 billion to US $7.4 billion by 2030 (16). Thus, DFU is associated with significant morbidity and mortality, as well as significant economic, social, and public health burdens.

Therefore, the treatment of DFU has become an urgent problem. Currently, the first-line routine treatment for DFU includes blood glucose control, conventional treatment (infection management, debridement, wound discharge, dressing), and angioplasty for ischemic peripheral artery disease (PAD) (17). However, the current treatment of DFU remains unsatisfactory. It has been reported that the median healing time of DFU without surgery is about 12 weeks (18), and about 20% of patients still have no healing after 1 year, with a recurrence rate of 40% in the same year (3). Therefore, the development of a fast, effective, and economical treatment for DFU is an important issue. In recent years, related studies have found that the use of stem cells or growth factors can form the basis of a new treatment, which can restore the body's normal healing process. Of these, PRP is of great interest because platelets possess a variety of growth factors, which are essential for tissue repair and regeneration, and have antibacterial properties in traumatic injuries (19, 20). PRP is a plasma preparation rich in platelets with a higher concentration than whole blood (21). The concentration of platelets in its plasma is above baseline, ranging from 150×103/dL to 400 x103/dL (22), which is 4-5 times higher than in whole blood (23, 24). The classic method of PRP preparation consists of two steps, the first step will be centrifugation to separate the blood components into three layers: a red blood cell layer, a light-colored coating layer (which contains most platelets and white blood cells) and poor quality platelet plasma, and the second step harvests concentrated platelets in a small volume of plasma, called PRP (25). The role of PRP in wound healing is mainly through the release of various bioactive molecules stored in platelets. In recent years, many studies have conducted relevant analysis on the efficacy of PRP in the treatment of DFU, but they are only limited to a few indicators, and there are still different conclusions, such as: Tasmania et al. concluded that the use of PRP in DFU promoted wound healing, reduced ulcer volume, reduced the time to complete wound healing, and reduced the incidence of adverse events, with no difference in the probability of wound complications (26). This is consistent with previous findings (27–31). However, Ajay et al. concluded that PRP had no significant effect on promoting ulcer healing (32). As one of the most important and common complications of diabetic patients, DFU has a profound impact on the prognosis, amputation and even death of patients. As a new method to treat DFU, the efficacy of PRP in DFU is worth further study. Therefore, the main purpose of this review is to review the results of various studies on the efficacy of PRP in the treatment of DFU.

## Review method

### Search strategy

This review was registered at the International Platform of Registered Systematic Review and Meta-analysis Protocols(INPLASY).The registration number was INPLASY2023110003.A literature search was undertaken in PubMed, CNKI, EMBASE, the Cochrane Library, the WanFang Database and the WeiPu Database by computer. The retrieval time was from the establishment of the database to June 2023, using the combination of subject terms and free words. The search terms included "Diabetes", "Diabetic foot ulcer", "Platelet-rich plasma", "Diabetic complications" and "Efficacy”. The search strategies for each database were presented in **Appendix 1**.

### Inclusion and exclusion criteria

The included studies were clinical trials (including randomized controlled trials、case-controlled trials、prospective observational) and retrospective studies (there were no language or location restrictions).We excluded case reports, letters, reviews. We included studies evaluating the efficacy of PRP in the treatment of DFU. Since the conventional first-line treatment of DFU includes blood glucose control, infection management, debridement, wound undressing, dressing, and vascular surgery for PAD (17), therefore, the relevant efficacy indicators included in our study mainly included wound healing rate, healing time, ulcer area reduction rate, ulcer recurrence rate, amputation rate or follow-up surgical treatment rate, infection rate, adverse event, length of stay, hospitalization cost, etc.

### Data extraction and quality evaluation

Two evaluators independently searched the database according to the inclusion and exclusion criteria, searched the full text of the initially included literatures, and extracted the literature data using a unified table, including the author's name, publication year, country, study type, research topic, number of studies, research time, main outcome indicators, and research conclusions. The included studies were evaluated from five aspects: randomization method, baseline comparability, intervention measures, blind method, and result analysis by using the Centre for Evidence-Based Medicine at Oxford University, UK. Evaluators made "yes", "no", "unclear" judgments for each evaluation item. We recorded the scores by using a scoring method ranging from 0 to 5, with 1 point for each project. The total score ≤2 points was considered as low-quality research, and ≥3 points was considered as high quality research.

### Statistical analysis

We used the Stata or R software for statistical analysis, using relative hazard (RR) and 95% confidence interval (CI) as the evaluation index of the results, represented by mean difference and 95% CI. First, heterogeneity was assessed using the X2 test (a=0.05) and a quantitative analysis of I2 for heterogeneity (I2 ≥ 50%) conducted. In cases of no heterogeneity between the research results, the meta-analysis was conducted. In cases of statistical heterogeneity between the research results, the source of heterogeneity was further analyzed, and the random heterogeneity model was used after excluding the influence of obvious clinical heterogeneity. Funnel maps created using the Stata software were employed to detect publication bias.

## Results

### Study selection

**Figure 1:**
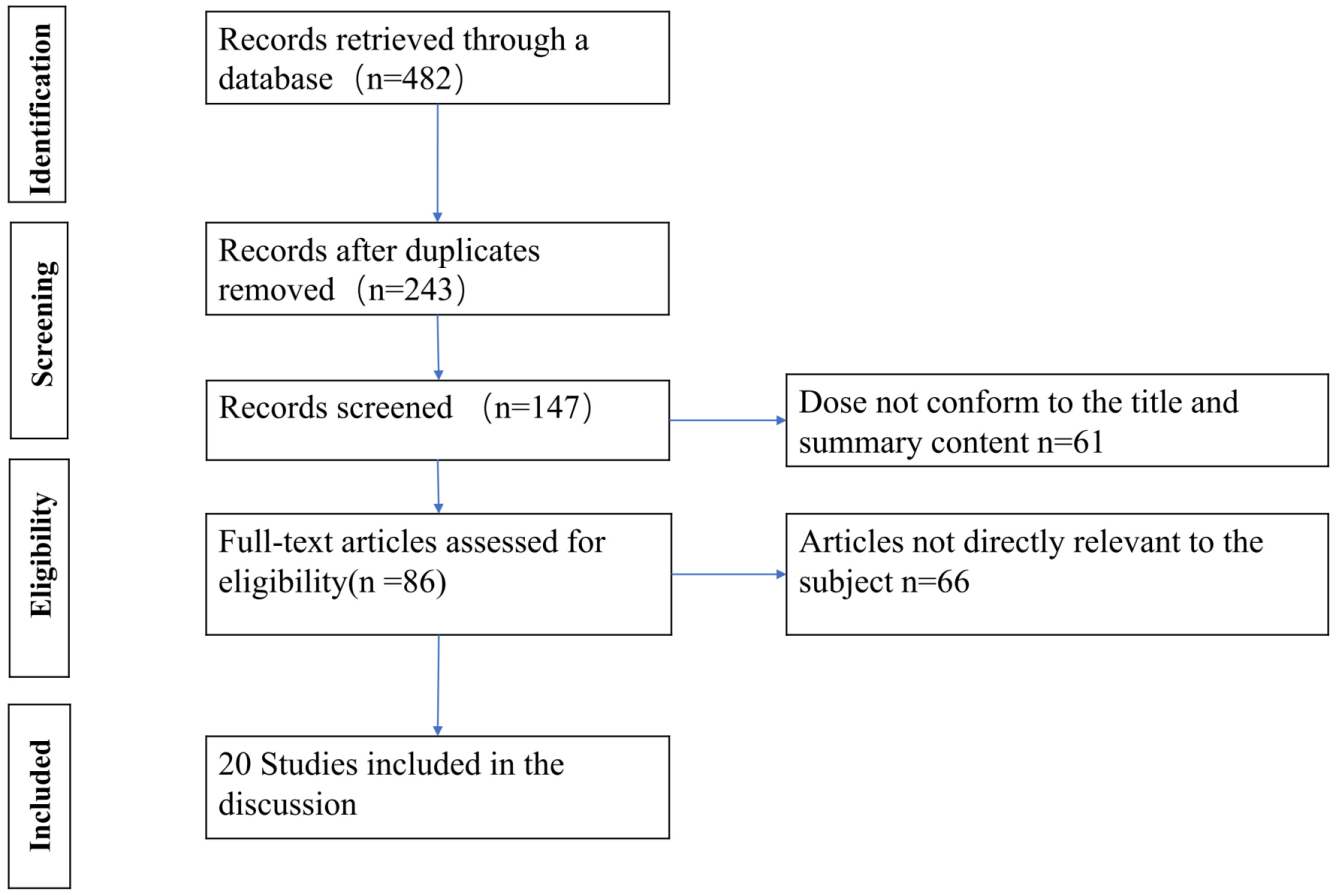
PRISMA flow diagram for the literature search and study selection.

According to the pre-designed literature search strategy, a total of 482 articles were retrieved by June 2023, and after removing duplicate studies, we found 243. Then after reviewing the title and abstract, another 96 articles were excluded and 147 articles were reviewed in detail, of which 86 were deemed likely to qualify for this review. Ultimately, we included 20 studies in this review. The remaining 66 studies were excluded due to a lack of data on the efficacy of PRP in DFU in the full text. We adhered to reporting and conduct guidance based on the Preferred Reporting Items for Systematic Review and Meta-Analysis (PRISMA) statement (**Figure 1**).

### Characteristics of studies

We summarized the basic characteristics of the included studies, as shown in **Table 1**. The included studies were published between 2005 and 2023, with five studies from Egypt (34, 38–40, 48), four from China (41, 44, 45, 52), three each from India (32, 37, 50) and Iran (35, 36, 51), two from the United Kingdom (33, 49), and one each from the United States (43), Pakistan (46), and Lithuania (42). The studies included seven different interventions: fat transplantation, fat transplantation +PRP, PRP, conventional therapy (standard care, saline dressing), hyperbaric oxygen therapy, stem cell transplantation, and stem cell transplantation +PRP. Among them, there were 14 studies comparing PRP with conventional treatment, 2 studies comparing fat transplantation, fat transplantation +PRP with conventional treatment, 1 study comparing different types of PRP, 1 study comparing hyperbaric oxygen and stem cell transplantation with PRP and conventional treatment respectively, and 1 study comparing the effect of PRP on diabetic feet with different wound sizes.

**Table 1:** Characteristics of Included Reports (n=20).

Study	Study design	Included subjects (treatment vs control)	Length of study	Wound aetiology	Main findings (treatment vs control)
Grant, UK, 2021 (33)	RCT	Fat grafting vs PRP+fat grafting vs Regular foot care (5 vs 6 vs 5)	4 weeks	DFU	Fat transplantation plus PRP can increase neovasculariz -ation and graft survival in diabetic foot ulcers.
Marwa, Egypt, 2016 (34)	RCT	PRP vs Antibacterial ointment dressing(28vs28)	12weeks	DFU	Autologous platelet gels are more effective than topical anti-inflammatories in the cure rate and prevention of infection in cleaning type 2 diabetic ulcers.
Ajay, India, 2021 (32)	RCT	Normal saline dressing vs PRP dressing (30 vs 30)	6 weeks or till complete healing of ulcer	DFU	There was no difference between PRP and normal saline dressing in the treatment of diabetic foot ulcer.
Nasser, Iran, 2021 (35)	RCT	Normal saline dressing vs PRP dressing (47vs43)	6 months	DFU	PRP can effectively promote the healing of foot ulcers in diabetic patients regardless of age, gender, smoking status or blood pressure status, and can shorten the healing time of DFUs.
Babaei, Iran, 2017 (36)	Prospective study	Group 1: wound size diameter of 2–5.5cm2 Group 2: wound size diameter of 5.5–8.5cm2 Group 3: wound size diameter of 8.5–12.5cm2. A total of 150 patients completed the study	4 weeks or till complete healing of ulcer	DFU	There was a significant difference in wound healing time related to ulcer size, with patients in the smallest ulcer group (2-5.5cm2) healing faster than those in the largest ulcer group (8.5-12.5cm2). In diabetic patients with large, non-healing ulcers, skin grafts must be used as a final method of wound healing.
Shailendra, India, 2018 (37)	Prospective study	Standard treatment vs PRP(26vs29)	4weeks	DFU	In this study, PRP was used and no adverse reactions were reported. The healing rate was better with PRP,
Ahmed, Egypt, 2019 (38)	RCT	Normal saline dressing vs PRP dressing	20 weeks	DFU	Using PRP gel as a dressing for chronic DFU resulted in a more significant reduction in ulcer size compared to conventional saline dressing. In addition, the time to reach the maximum possible healing point with the smallest wound size was significantly shortened when PRP was used as a dressing regimen.
Yasser,Egypt, 2022 (39)	RCT	Normal saline dressing vs PRP dressing(36vs36)	20 weeks	DFU	Higher wound healing rates were achieved in a shorter period of time using auto PRP.
Hossam, Egypt, 2022 (40)	Prospective study	Standard treatment vs PRP (40vs40)	12weeks	DFU	PRP can accelerate wound healing in DFU and reduce local infection rate.
Xie, China, 2019 (41)	RCT	Standard treatment vs APG (23vs25)	8weeks	DFU	APG can accelerate the healing of diabetic ulcer sinus, shorten the healing time of clinical difficult wounds, shorten hospital stay and reduce hospitalization costs. APG can also accelerate the transformation of bacterial culture from positive to negative, which has certain antibacterial effect.
Domantas, Lithuania, 2019 (42)	Prospective randomized controlled trial	Standard treatment vs PRP (34vs35)	8 weeks	Chronic leg ulcer	APG applied locally to leg ulcers of various etiologies can reduce the size of the wound and induce granulation tissue formation.
Vickie, USA, 2006 (43)	RCT	Normal saline dressing vs PRP dressing(36vs36)	12weeks or till complete healing of ulcer	DFU	Wounds treated with PRP gel healed significantly more easily than those treated with control gel.
Li, China, 2014 (44)	Prospective randomized controlled trial	Standard treatment vs APG (58vs59)	12weeks	DFU	Topical application of APG plus standard in the treatment of diabetic refractory skin ulcers, including diabetic foot ulcers, has been shown to be safe and effective. It can improve the grade of wound healing, shorten the healing time, speed up the healing speed, but does not cause systemic or local side effects.
Li, China, 2022 (45)	Retrospective study	Standard treatment vs APG (36 vs 36)	The wound healed or at the end of the 12th week.	DFU	After treatment, there were statistically significant differences in healing time, hospital stay, healing rate and surface area reduction between the two groups.
Asad,Pakistan, 2022 (46)	Prospective study	Standard treatment vs PRP (40vs 40)	180 ds	DFU	The effect of PRP in the treatment of diabetic foot ulcer is obviously better than that of conventional dressing.
He, China, 2020 (47)	Prospective case-control study	Standard treatment vs Al-PRP vs Au-PRP (30 vs 20 vs 25)	Until wound closure was complete or until surgical operation, even amputation.	DFU	Both al-PRP and au-PRP can effectively and safely promote wound healing in patients.
Hany, Egypt, 2011 (48)	RCT	Platelet-poor plasma vs PRP (12vs 12)	20 weeks, or stopped whenever healing occurred	Chronic diabetic ulcers	PRP promotes healing of chronic diabetic foot ulcers.
Smith, UK, 2020 (49)	RCT	Standard care(6)/Fat grafting (6)/PRP+Fat grafting (6)	12 weeks .	DFU	Adipose-derived stem cells are present in fat grafts, and mixing them with platelet-rich plasma (PRP) improves graft survival.
Sachin, India, 2013 (50)	A prospective randomized trial	Standard care(20)/ HBOT(20)/PRP(20	–	DFU	Diabetic foot ulcer management requires multidisciplinary and aggressive approach.PDGF should be recommended for all grade III and V diabetic foot ulcer at least 8 weeks old. HBO is equally good an option.
Meamar, Iran, 2021 (51)	RCT	Standard care(7)/SCs (11)/SCs+PRP(10)	16 weeks	DFU	Stem cells combined with PRP promote neovasculariz -ation.

PRP, Platelet-rich plasma; APG, Autologous platelet-rich gel; VLUs, Venous leg ulcers; DFU, Diabetic foot ulcer; RCT, Randomized Controlled Trial; Al-PRP, Allogeneic platelet-rich plasma; Au-PRP, Autogenous platelet-rich plasma; HBOT, hyperbaric oxygen therapy; SCs, stem cells.

After quality evaluation, 19 studies scored ≥3 points, and only 1 study scored ≤2 points, which was good included in the study quality. The results were shown in **Table 2**. Many studies failed to score in the category of blinded or not, possibly because the study of DFU in the PRP group was an open-label study, both the patient and the investigator knew the nature of the study and the assignment of the study group, so a blind approach could not be implemented.

**Table 2:** The quality assessments of studies.

Author, country and published year	①	②	③	④	⑤	Score
Grant,UK,2021 (33)	Yes	Yes	Yes	No	Yes	4
Marwa,Egypt,2016 (34)	Yes	Yes	Yes	No	Yes	4
Ajay,India,2021 (32)	Yes	Yes	Yes	No	Yes	4
Nasser,Iran,2021 (35)	Yes	Yes	Yes	No	Yes	4
Babaei,Iran,2017 (36)	No	No	Yes	No	Yes	2
Shailendra,India,2018 (37)	Unclear	Yes	Yes	No	Yes	3
Ahmed,Egypt,2019 (38)	Yes	Yes	Yes	No	Yes	4
Yasser,Egypt,2022 (39)	Yes	Yes	Yes	No	Yes	4
Hossam,Egypt,2022 (40)	Yes	Yes	Yes	No	Yes	4
Xie,China,2019 (41)	Yes	Yes	Yes	No	Yes	4
Domantas,Lithuania.,2019 (42)	Yes	Yes	Yes	No	Yes	4
Vickie,USA,2006 (43)	Yes	Yes	Yes	No	Yes	4
Li,China,2014 (44)	Yes	Yes	Yes	No	Yes	4
Li,China,2022 (45)	Unclear	Yes	Yes	No	Yes	3
Asad,Pakistan,2022 (46)	Yes	Yes	Yes	No	Yes	4
He,China,2020 (47)	Unclear	Yes	Yes	No	Yes	3
Hany,Egypt,2011 (48)	Yes	Yes	Yes	No	Yes	4
Smith,UK,2020 (49)	Yes	Yes	Yes	No	Yes	4
Sachin,India,2013 (50)	Yes	Yes	Yes	No	Yes	4
Meamar,Iran,2021 (51)	Yes	Yes	Yes	No	Yes	4

①Whether the random grouping method is really adopted

②Whether the groups were comparable at baseline.

③In addition to the interventions to be validated, were other treatments and care measures received by the groups the same?

④Were the subjects and outcome evaluators blinded?

⑤Were all participants included in the results analysis?

### Overview of the efficacy of PRP in the treatment of DFU

A total of 63 measures of efficacy of PRP for diabetic foot were reported (**Table 3**). By eliminating the duplicate measures reported in the study, we finally introduced 19 measures for the evaluation of the efficacy of PRP in DFU treatment. The efficacy measures that were repeated in various studies mainly included the rate of complete ulcer healing, the percentage of ulcer area reduction, the time required for ulcer healing, wound complications (including infection rate, amputation rate, and degree of amputation), the rate of ulcer recurrence, and the cost and duration of hospitalization for DFU, as well as subsequent survival and quality of life scores. Among these studies, twelve studies reported the complete healing rate of DFU patients after treatment, the results of meta-analysis showed that the use of PRP resulted in significantly higher complete-healed DFU compared to conventional treatment (OR = 4.37, 95% CI 3.02-6.33, P < 0.001) as shown in **Figure 2.1**. Five studies reported that the healing time of patients with DFU treated using PRP was significantly shorter compared to conventional treatment (MD=-3.21, 95% CI -3.83 to -2.59,P < 0.001)as shown in **Figure 2.2**. Four studies reported that the ulcer area of patients with DFU treated using PRP was no significantly reduced compared to conventional treatment(MD=5.67, 95% CI -0.77 to 12.11,P =0.08>0.05 )as shown in **Figure 2.3**. **Figure 3** shows the funnel diagram after adjustments for comparison. Most studies on the funnel plot are symmetrically distributed on both sides of the vertical line at X = 0, indicating no significant publication bias.

**Table 3:** Primary outcome measures of PRP treatment for DFU.

Author, country and published year	Primary outcome measures
Grant,UK,2021 (33)	The formation of new blood vessels Graft survival rate
Marwa,Egypt,2016 (34)	Ulcer healing rate Wound infection rate
Ajay,India,2021 (32)	Ulcer healing rate Percentage reduction in ulcer area
Nasser,Iran,2021 (35)	The time for the ulcer to heal Frequency and degree of amputation Whether further treatment is needed, such as tissue transplantation or angioplasty.
Babaei,Iran,2017 (36)	Ulcer healing rate Percentage reduction in ulcer area The time for the ulcer to heal Ulcer recurrence rate
Shailendra,India,2018 (37)	Wound score Ulcer healing rate The time for the ulcer to heal Wound infection rate
Ahmed,Egypt,2019 (38)	Percentage reduction in ulcer area The time for the ulcer to heal Ulcer healing rate Wound complication
Yasser,Egypt,2022 (39)	Ulcer healing rate The time for the ulcer to heal
Hossam,Egypt,2022 (40)	Ulcer healing rate The time for the ulcer to heal Wound infection rate Amputation Hospitalization expenses
Xie,China,2019 (41)	Ulcer healing rate Wound infection rate Sinus closure rate Time in the hospital Hospitalization expenses
Domantas,Lithuania.,2019 (42)	Ulcer healing rate Wound infection rate Percentage reduction in ulcer area
Vickie,USA,2006 (43)	Ulcer healing rate
Li,China,2014 (44)	Grade of wound healing The rate at which wounds heal The time for the ulcer to heal Adverse reaction to the wound Recurrence rate of ulcers Survival rate
Li,China,2022 (45)	The time for the ulcer to heal Ulcer healing rate Percentage reduction in ulcer area Time in the hospital Adverse reaction to the wound
Asad,Pakistan,2022 (46)	Ulcer healing rate
He,China,2020 (47)	Ulcer healing rate The time for the ulcer to heal Adverse reaction to the wound
Hany,Egypt,2011 (48)	Ulcer healing rate
Smith,UK,2020 (49)	Percentage reduction in ulcer area Ulcer healing rate The wound pressure score Hospitalization expenses Adverse reaction to the wound Quality of life score
Sachin,India,2013 (50)	Ulcer healing rate
Meamar,Iran,2021 (51)	Percentage reduction in ulcer area Ulcer healing rate The formation of new blood vessels

**Figure 2:**
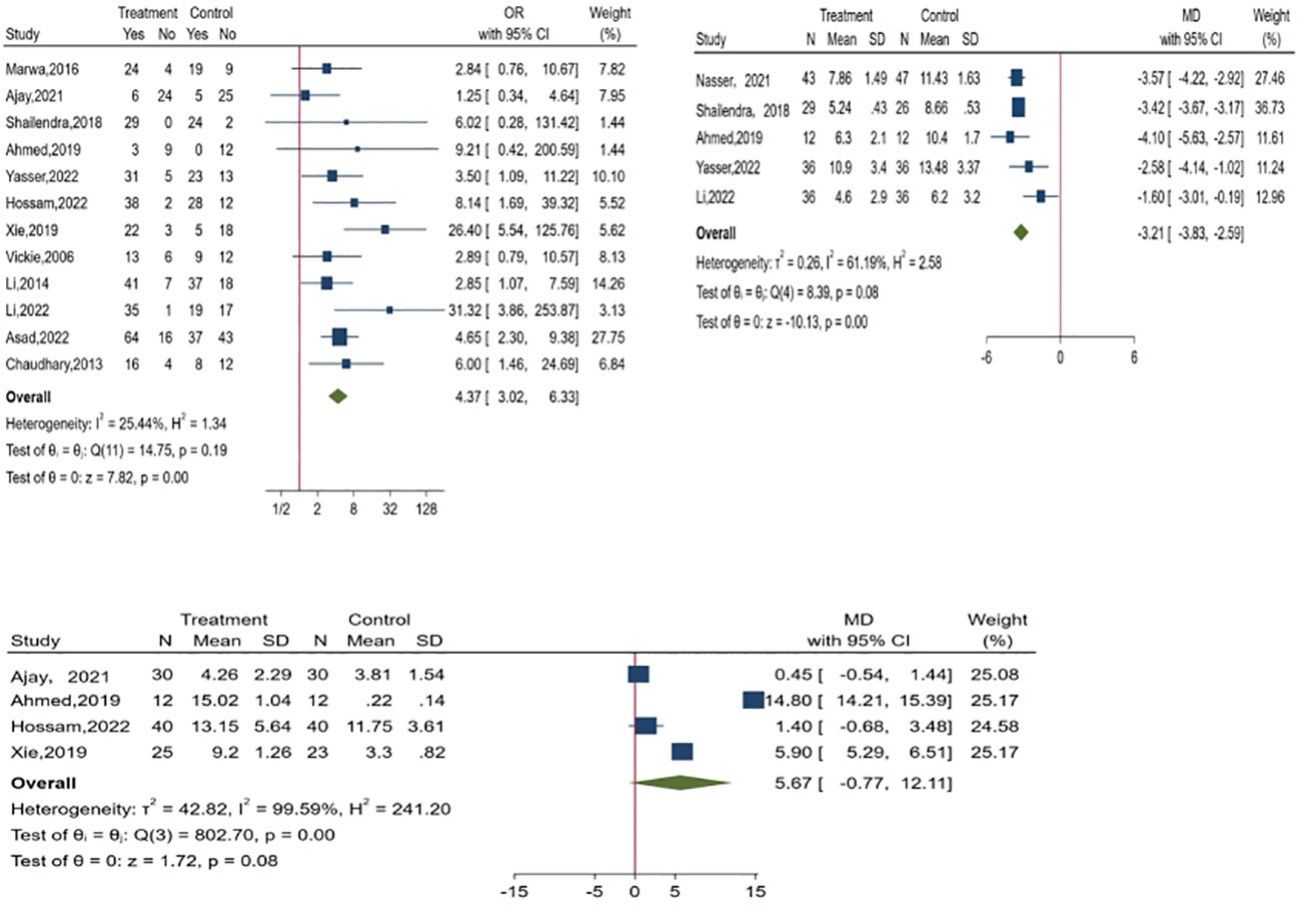
Forest plot of the effect of PRP compared with conventional treatment of DFU.

**Figure 3:**
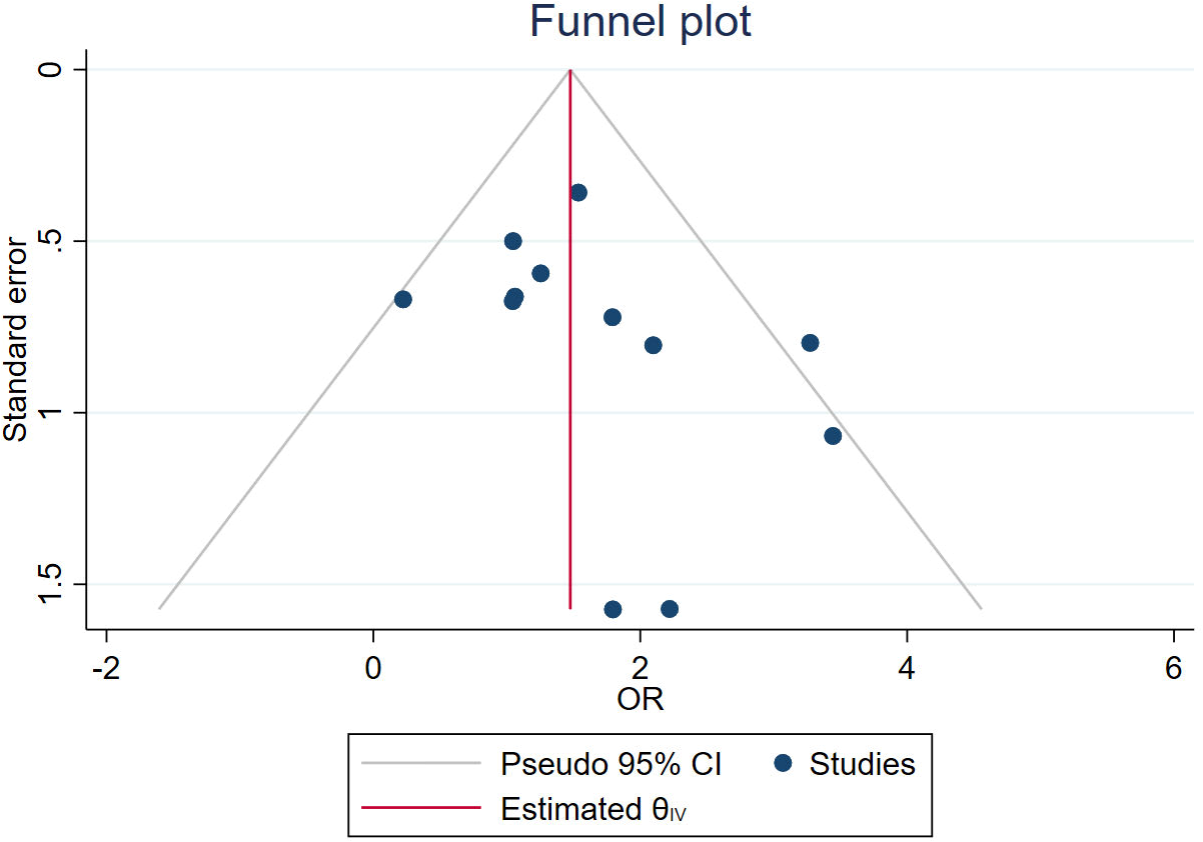
The funnel plot.

## Discussion

DFU is one of the most common, serious and costly complications of diabetes and the leading cause of hospitalization for people with diabetes worldwide (53). DFU is also the main cause of lower limb amputation in diabetic patients, which often leads to disability (54, 55), emotional disorders, socio-economic problems, and severely impaired quality of life, and even death in severe cases (56, 57). Studies have found that about 15%-25% of people with diabetes have experienced DFU during their lifetime (3, 57–59).

Through meta-analysis, we found that compared with conventional or standard care, the use of PRP in DFU can effectively improve the ulcer healing rate, and shorten the ulcer healing time, which is consistent with the results of previous studies (34, 37–40, 43, 46, 48). However, there was no significant difference in reducing the increase of ulcer area, which may be due to the small number of included study data and large heterogeneity.

In addition, a study has found that PRP can still effectively promote the healing of DFU and shorten the healing time after excluding factors such as age, gender, smoking status and blood pressure status of diabetic patients, but had no significant effect on reducing the need for amputation, the level of amputation, or the need for further treatment (such as graft or angioplasty) (35). He et al. divided PRP into autogenous PRP(au-PRP)and allogeneic PRP(al-PRP), and found that PRP in both groups could effectively and safely promote wound healing in DFU compared with conventional dressing treatment, suggesting that al-PRP could be used as a ready solution for DFU when au-PRP was limited (45). The efficacy of PRP is also different among ulcers of different sizes. Babaei et al. 's prospective study grouped diabetic foot wounds according to the size of ulcers and found a significant difference in wound healing time, which was related to the size of ulcers. Patients with the smallest ulceration group (2-5.5cm2) had faster wound healing time than those with the largest ulceration group (8.5-12.5cm2).According to this study, PRP is not recommended for large, non-healing ulcers in DFU, and skin graft must be used as the final method of wound healing (36). In the long-term follow-up of patients, Li et al. found that there was no significant difference in the long-term recurrence rate of ulcers between the PRP group and the control group (44). We collected meta-analyses related to PRP treatment of skin ulcers in the past 5 years and summarized them, as shown in **Table 4**. The results showed that PRP had a positive effect on promoting ulcer healing in the treatment of chronic skin ulcers, including DFU, venous ulcers of lower extremities and pressure ulcers. In two recent meta-analyses, Gong and Peng et al. found that PRP treatment of DFU increased the possibility of wound healing, reduced the ulcer volume, and reduced the time of complete wound healing (28, 63). This is consistent with the results of our meta-analysis. Secondly, the meta-analysis of Tasmania also showed that in terms of safety, platelet-rich plasma and standard treatment had no difference in the probability of wound complications or recurrence, but overall reduced the incidence of adverse events (26). These results are consistent with the findings of the meta-study by Dai,Qu (27–31, 62). Although there is no same conclusion about whether or not DFU treated by PRP has the same effect on patients' later amputation and the degree of amputation, it has been found in related studies that the overall amputation rate of DFU patients in PRP group is lower (62). PRP alone is used to treat DFU, which can effectively increase the healing rate of ulcers and shorten the healing time. In addition, relevant studies have also found that when PRP is combined with other treatment modalities, it can also work to a certain extent. Some studies reported that the addition of PRP to fat grafts resulted in increased angiogenesis in fat grafts and thus improved the viability of fat graft cells (33, 47, 49, 52, 60, 61, 64–67). In another study, the addition of PRP to mesenchymal stem cells also observed significant neovascularization and more significant wound reduction (51). Yin et al. found that VSD combined with PRP could also significantly shorten the healing time and improve the healing rate of ulcers (68).

**Table 4:** Meta-analysis of PRP treatment for chronic wounds.

Author and published year	Study design	Included subjects (treatment vs control)	Number of included studies	Wound aetiology	Main findings (treatment vs control)
Tasmania, 2018 (26)	Meta-Analysis	Platelet-rich plasma vs standard treatment or any other alternative therapy.	Eight randomized clinical trials and two prospective longitudinal-observational studies	DFU	Platelet-rich plasma therapy increases the likelihood of chronic wound healing, and ulcer volume and wound full healing time decrease. In terms of safety, platelet-rich plasma did not differ from standard treatment in the incidence of wound complications, but reduced the incidence of adverse events.
Dai, 2020 (27)	Meta-Analysis	Platelet-rich plasma vs standard care or conventional conservative therapy	10 RCTs with 456 patients	DFU	Autologous PRP can improve the rate of complete ulcer healing and shorten the healing time without increasing the incidence of adverse events.
Qu, 2021 (31)	Meta-Analysis	Platelet-rich plasma vs any other wound care without PRP	20 RCTs and Five observational studies.	Lower-extremity diabetic ulcers; Lower-extremity venous ulcers; Pressure ulcers.	PRP treatment significantly improved the wound closure of lower limb diabetic ulcer, shortened the wound closure time, and reduced the wound area and depth.
Gong, 2022 (28)	Meta-Analysis	Platelet-rich plasma vs standard management	19 Studies (1435 subjects with diabetic foot ulcer wounds at the baseline of the studies; 723 of them were treated with platelet-rich plasma, and 712 used control)	DFU	Autologous and allogeneic platelet-rich plasma can significantly improve the rate of complete healing of diabetic foot ulcers.
Xia, 2019 (29)	Meta-Analysis	Platelet-rich plasma VS standard wound care for chronic wounds.	15 RCTs with 630 adult patients	Chronic nonhealing Ulcers	The overall healing rate was significantly higher and faster in the platelet-rich plasma group.
LI, 2019 (60)	Meta-Analysis	APG vs Standard care/conventional treatment,	15 RCTs with 829 patients	Diabetic chronic cutaneous ulcers(such as leg ulcers, foot ulcers, back ulcers, hip ulcers and so on)	APG significantly improved the healing rate, shortened the healing time and reduced the incidence of infection. APG treatment can shorten the length of hospital stay, reduce hospitalization costs to some extent, and do not increase the occurrence of adverse events.
Shen, 2019 (61)	Meta-Analysis	PRP VS Conventional treatments	19 RCTs with 909 patients)	Diabetic ulcers, vascular ulcers, and pressure ulcers	PRP achieved higher cure rates, higher percentage of area reduction, and smaller final area in vascular ulcers. However, this advantage disappeared in diabetes and pressure ulcers.
Kaissar, 2022 (30)	Meta-Analysis	PRP VS Standard care	Ten prospective studies (8 randomized)	Venous leg ulcers (VLUs)	PRP has significant beneficial effects in terms of healing rate, surface reduction and healing time reduction.
Peng, 2023 (62)	Meta-Analysis	PRP VS stanconventional treatments,	A total of 10 RCTs involving 550 patients	DFU	Compared to conventional treatment, PRP effectively promoted the healing of patients with DFU by evidently improving the healing rate and healing time.

The activity of PRP is related to many factors in the preparation process, which to some extent affects the efficacy of PRP in the treatment of diabetic foot. We summarized the factors related to PRP preparation in the included studies, as shown in **Table 5**. We can find that an important factor affecting the preparation of PRP is the centrifuge conditions, such as force and duration, which are significantly different in different studies. Another important factor is the difference in activators, and different activators may affect the release of bioactive molecules and the cleavage of fibrinogen. In addition, we can see that PRP preparation is mostly extracted from patients' peripheral blood, and there are individual differences among different patients, which may also cause different PRP activities prepared under the same preparation conditions.

**Table 5:** Factors related to PRP preparation in the study.

Author, country and published year	Blood collection volume	Anticoagulant	Centrifugal condition	Activator
Marwa, Egypt, 2016 (34)	20 ml of peripheral blood	–	the first cycle was at 1.500 rpm for 5 minutes, the second cycle at 3.500 rpm for 5 minutes	2 ml of thrombin and 2 ml of 10% calcium chloride
Ajay, India, 2021 (32)	20 ml of blood	Sodium citrate	the blood is centrifuged in a centrifuge at 2,500 revolutions per minute (rpm) for five minutes	–
Nasser,Iran, 2021 (35)	20 ml of peripheral venous blood	An anticoagulant (citrate dextrose)	the first cycle was at 1.500 rpm for 5 minutes,the second cycle at 3.500 rpm for 5 minutes	0.2 ml thrombin for each mL of PRP and calcium chloride
Babaei,Iran, 2017 (36)	blood (30-40ml)	3.8% sodium citrate	the first cycle was at 1400-1800 rpm for 10-12 minutes,the second cycle at 2500rpm for 10-15 minutes.	20mM calcium chloride solution (CaCl2) in a ratio of 1 CaCl2:5PRP
Shailendra, India, 2018 (37)	up to 20ml, with volume of blood taken dependent on size of the wound	anticlotting agent (ACD-A Anticoagulant Citrate Dextrose Solution, Solution A, USP [2.13% free citrate ion])	2000-3200rpm for 10-15 minutes	calcium chloride or thrombin (0.2ml CACl2 :1ml PRP)
Ahmed,Egypt, 2019 (38)	20ml of venous blood	citrate dextrose	two cycles: the first cycle was at 3600 rounds/ min,the second cycle at 2400 rounds/ min	20% calcium chloride
Yasser,Egypt, 2022 (39)	20 ml of blood	anticoagulant sodium citrate.	the first spin was at a speed of 1000 rpm for 10 minutes, and the second spin was at a speed of 3000 rpm for another 10 minutes.	calcium gluconate 10%; every 9 mL was activated by adding 1 mL of calcium gluconate
Hossam,Egypt ,2022 (40)	50ml of venous blood	anticoagulant, i.e. citrate dextrose A	the first spin was at a speed of 1,000 rpm for 10 min, and the second spin was at a speed of 1,500 rpm for 10 min	CaCl2 at 10%
Xie,China, 2019 (41)	20ml of venous blood	potassium citrate.	the first spin was at a speed of 2000 rpm for 4 min, and the second spin was at a speed of 4000 rpm for 6 min	calcium chloride and thrombin
Domantas, Lithuania., 2019 (42)	8ml of venous blood	–	using a certified system of medical devices, RegenKitBCT (RegenLab, Switzerland)	–
Vickie,USA, 2006 (43)	20ml of venous blood	–	a small, portable centrifuge for 1.5 minutes	–
Li,China, 2014 (44)	20-100 ml (based on the wound sizes) peripheral venous blood	–	the first spin was at a speed of 313 × g for 4 minutes, and the second spin was at a speed of 1252 × g for 6 minutes	thrombin and calcium gluconate in a proper proportion of 10:1
Li,China, 2022 (45)	the amount of blood was determined according to the size of the ulcer surface (10 ml/1 cm2)	3.2% sodium citrate	the first spin was at a low speed of 2000 r/min for 5 minutes, and the second spin was at a speed of 1200 r/min for 10 minutes	thrombin and 10%calcium gluconate in a proper proportion of 10:1
He,China, 2020 (47)	50 to 100 ml (based on the wound sizes) of peripheral venous	acid citrate dextrose solution ,B anticoagulants	600 rpm for 15 min. 1,135 g for 7 min	thrombin and 10%calcium gluconate in a proper proportion of 10:1
Hany,Egypt, 2011 (48)	–	anticoagulant (citrate dextrose)	the first centrifugation is called 'soft spin' (1007 g) the second centrifugation (447-5g) called 'hard spin'	thrombin (0-2 ml for every 1 cc PRP) and calcium chloride 10% (0-1 ml)
Smith,UK, 2020 (49)	52 ml of venous blood	adenosine citrate dextrose acid (ACD-A)	using the Food and Drug Administration-approved and CE-marked Angel PRP processing device (Arthrex, Naples, Florida)	–

PRP for diabetic foot can effectively reduce the ulcer area, improve the ulcer healing rate, and to a certain extent reduce the infection rate of the wound and reduce the occurrence of complications. The reason why PRP can play such a curative effect is on the one hand due to the particularity of DFU healing, on the other hand, it depends on the mechanism of PRP. Changes in the micro-environment due to diabetes mellitus (DM) alter normal cell recruitment and activation and lead to impaired or delayed wound healing (69–71). In this way, the wound is disconnected from the normal process and does not heal for a long time, resulting in the formation of diabetic chronic refractory ulcers. At present, it is generally believed that peripheral neuropathy and peripheral vascular disease are the two main factors causing foot ulcers in diabetic patients (72). Secondly ,the hyperglycemic environment in diabetics also leads to increased production of advanced glycation end products (AGEs) and continued elevated levels of inflammatory cytokines (i.e., interleukin (IL-1ß) and tumor necrosis factor a (TNF-a), thus impedes the normal wound healing process (73, 74). At the same time, hyperglycemia also leads to an increased risk of infection, and the rapid spread of infection and high microbial burden also adversely affect the wound healing process. In addition, inflammation disorders are also a hallmark of diabetes and underlie many complications of diabetes, including diabetic ulcers (75). Given these pathological abnormalities present in diabetes, it is recommended that specific synthetic growth factors be used topically to manage diabetic foot ulcer wounds. PRP's effect on wound healing is mainly through the release of various bioactive molecules stored in platelets, including PDGF, transforming growth factor β(TGF-β), VEGF, epithelial growth factor (EGF), and adhesion molecules such as fibrin, fibronectin, and hyalenin (76, 77). These factors are known to regulate processes such as cell migration, attachment, proliferation, and differentiation, and to play an important role in wound healing and regeneration by binding to specific cell surface receptors to promote the accumulation of extracellular matrix(ECM) (76, 78–80). In addition to growth factors, PRP include many important proteins, such as fibrin, which not only provide scaffolds for tissue regeneration, but also promote wound contraction, blood clotting, and wound closure (81, 82). In addition, activated PRP contains a variety of antibacterial proteins. Previous studies have shown that activated PRP can inhibit staphylococcus aureus, staphylococcus epidermidis, escherichia coli, klebsiella pneumoniae, and methicillin-resistant staphylococcus aureus , without drug resistance, and has synergistic effects with antibacterial agents (83–85). The combination of these action characteristics makes PRP more promising than conventional antibiotic prescribing (85).

## Limitations

In this study, employed a comprehensive search strategy for key review tasks that contains all of the studies that assessed the efficacy evaluation of PRP for DFU, however, it is possible that some unpublished data were missed. Second, there are too few data on some indicators of the efficacy of PRP in DFU treatment (such as amputation rate, degree of amputation, and need for further treatment (tissue transplantation or angioplasty), so more clinical data are needed for further study.

## Conclusion

PRP can release various bioactive molecules and antibacterial proteins, which makes it effective in improving ulcer healing rate and shortening ulcer healing time when used in DFU. In the future, more studies are needed to further explore the efficacy of PRP. Secondly, the efficacy of PRP in the treatment of DFU is largely affected by various factors in the preparation process, so a standardized preparation process is essential.

## Author contributions

HO: Investigation, Methodology, Writing – original draft, Writing – review & editing. YT: Writing – review & editing. FY: Writing – review & editing. XR: Data curation, Writing – review & editing. JY: Methodology, Writing – review & editing. HC: Formal Analysis, Writing – review & editing. YY: Validation, Writing – review & editing.
