# Supplementary material for: Platelet-rich plasma for the treatment of diabetic foot ulcer: a systematic review
Source: Front Endocrinol (Lausanne). 2023 Nov 18;14:1256081. doi: 10.3389/fendo.2023.1256081 (PMC10760804; doi:10.3389/fendo.2023.1256081)
Supplement: Supplementary file 1 [file DataSheet_1.docx]

**Appendix 1. Search strategies of each database**

The following database will be searched from inception to 1, June, 2023.

**Database 1 Pubmed**

1 Diabetic foot ulcer[Mesh]

2 Diabetic foot ulcer [Title/Abstract] OR Diabetic foot [Title/Abstract] OR Diabetie foot

[Title/Abstract] OR Diabetes foot [Title/Abstract] OR Diabetes feet [Title/Abstract] OR

Diabitic foot [Title/Abstract] OR Diabetic patients with df [Title/Abstract] OR DFU

[Title/Abstract]

3 1 OR 2

4 Platelet-rich plasma [Mesh]

5 Platelet-rich plasma[Title/Abstract] OR Platelet rich plasma[Title/Abstract]OR Autologous platelet rich plasma[Title/Abstract] OR Allogeneic platelet-rich plasma[Title/Abstract] OR PRP[Title/Abstract] OR Au-PRP[Title/Abstract] OR Al-PRP[Title/Abstract]

6 4 OR 5

7 Observational study[Mesh]

8 Clinical trials[Title/Abstract] OR Randomized controlled trial[Title/Abstract] OR Retrospective study[Title/Abstract] OR Prospective study[Title/Abstract]

9 7 OR 8

10 3 AND 6 AND 9

**Database 2 China National Knowledge Infrastructure (CNKI)**

SU=('糖尿病' +'糖尿病足'+'慢性溃疡') AND SU=('自体富血小板血浆'+'异体富血小板血浆') AND SU=('疗效' )=AND SU= ('临床试验'+'回顾性研究')

**Database 3 EMBASE**

1 'Diabetic foot ulcer'/exp

2 'Diabetic foot ulcer':ab,ti OR 'Diabetic foot':ab,ti OR ' Diabetie foot':ab,ti OR 'Diabetes foot':ab,ti OR 'Diabetes feet':ab,ti OR 'Diabitic foot':ab,ti OR 'Diabitic foot':ab,ti OR 'DFU

':ab,ti

3 1 OR 2

4 'Platelet-rich plasma'/exp

5 'Platelet-rich plasma':ab,ti OR 'Platelet rich plasma':ab,ti OR 'Autologous platelet rich plasma':ab,ti OR 'Allogeneic platelet-rich plasma':ab,ti OR 'PRP':ab,ti OR 'Al-PRP':ab,ti OR 'Au-PRP':ab,ti

6 4 OR 5

7 'Observational study '/exp

8 'Clinical trials':ab,ti OR 'Randomized controlled trial':ab,ti OR 'Retrospective study':ab,ti OR 'Prospective study:ab,ti

9 7 or 8

10 3AND 6 AND 9

**Database 4 Cochrane Library**

1 Mesh descriptor: [Diabetic foot ulcer]explode all trees;

2 Diabetic foot ulcer:ti,ab,kw or Diabetic foot:ti,ab,kw or Diabetie foot:ti,ab,kw or Diabetes foot:ti,ab,kw or Diabetes feet:ti,ab,kw or Diabitic foot:ti,ab,kw or Diabetic patients with df :ti,ab,kw or DFU:ti,ab,kw (Word variations have been searched);

3 1 or 2

4 Mesh descriptor: [Platelet-rich plasma] explode all trees;

5 Platelet-rich plasma:ti,ab,kw or Platelet rich plasma:ti,ab,kw or Autologous platelet rich plasma:ti,ab,kw or Allogeneic platelet-rich plasma:ti,ab,kw or PRP:ti,ab,kw or Au-PRP:ti,ab,kw or Al-PRP:ti,ab,kw (Word variations have been searched);

6 7 or 8

9 Mesh descriptor: [Observational study] explode all trees;

10 Clinical trials:ti,ab,kw or Randomized controlled trial:ti,ab,kw or Retrospective study:ti,ab,kw or Prospective study:ti,ab,kw (Word variations have been searched);

11 9 or 10

12 3 and 6 and 9

**Database 5 Wanfang Database**

(糖尿病or糖尿病足or 慢性溃疡) and (富血小板血浆or自体富血小板血浆or异体富血小板血浆) and (疗效or 伤口愈合率or 愈合所需时间or溃疡面积减少率or溃疡复发率or截肢率or后续手术治疗率or感染率or不良反应事件)and (观察性研究or临床试验or随机对照实验or回顾性研究or前瞻性研究)

**Database 6 WeiPu Database**

(U=糖尿病 OR U=糖尿病足 OR U=慢性溃疡) AND (U=富血小板血浆 OR U=自体富血小板血浆 OR 异体富血小板血浆) AND (U=疗效 OR U=伤口愈合率 OR U=愈合所需时间 OR U=溃疡面积减少率 OR U=溃疡复发率 OR U=截肢率 OR U=后续手术治疗率OR U=感染率 OR U=不良反应事件)
